# Levels of infants’ urinary arsenic metabolites related to formula feeding and weaning with rice products exceeding the EU inorganic arsenic standard

**DOI:** 10.1371/journal.pone.0176923

**Published:** 2017-05-04

**Authors:** Antonio J. Signes-Pastor, Jayne V. Woodside, Paul McMullan, Karen Mullan, Manus Carey, Margaret R. Karagas, Andrew A. Meharg

**Affiliations:** 1 Institute for Global Food Security, Queen’s University Belfast, David Keir Building, Belfast, Northern Ireland, United Kingdom; 2 Department of Epidemiology, Geisel School of Medicine, Dartmouth College, 1 Medical Center Dr, 7927 Rubin Bldg, Lebanon, NH, United States of America; 3 UKCRC Centre of Excellence for Public Health, Institute of Clinical Science B, Royal Victoria Hospital, Belfast, Northern Ireland, United Kingdom; Stony Brook University, Graduate Program in Public Health, UNITED STATES

## Abstract

Early childhood inorganic arsenic (i-As) exposure is of particular concern since it may adversely impact on lifetime health outcomes. Infants’ urinary arsenic (As) metabolites were analysed in 79 infants by inductively coupled plasma—mass spectrometric detection (IC-ICP-MS) to evaluate i-As exposure pre- and post-weaning. Levels of i-As in rice-based weaning and infants’ foods were also determined to relate to urinary As levels. Higher As levels, especially of monomethylarsonic acid (MMA) and dimethylarsinic acid (DMA), were found in urine from formula fed infants compared to those breastfed. Urine from infants post-weaning consuming rice-products resulted in higher urinary MMA and DMA compared to the paired pre-weaning urine samples. The European Union (EU) has regulated i-As in rice since 1^st^ January 2016. Comparing infants’ rice-based foods before and after this date, little change was found. Nearly ¾ of the rice-based products specifically marketed for infants and young children contained i-As over the 0.1 mg/kg EU limit. Efforts should be made to provide low i-As rice and rice-based products consumed by infants and young children that do not exceed the maximum i-As level to protect this vulnerable subpopulation.

## Introduction

Early-life exposure to i-As, even at relatively low concentrations, is of particular concern due to infants and young children’s specific vulnerability to the adverse health effects of this group I human carcinogen, which may impact health and disease development throughout their lifespan, including neurological, cardiovascular, respiratory and metabolic outcomes [1–5]. Diet dominates i-As exposure when low i-As drinking water is available, especially for infants and young children due to their higher food consumption per body weight unit, and low dietary diversity dominated by breast milk or formula during the first 4–6 months followed by the introduction of solid foods such as rice-based products [6–10]. Breast milk contains low i-As concentrations, even in areas with high drinking water i-As, and thus exclusively breastfeeding has been suggested to protect infants from i-As exposure [8,11]. Formula powder can contain relatively low concentrations of i-As, and water used to mix the powdered formula may add significantly to the final concentration in the ready-made products. Non-dairy formulas contain higher i-As compared to dairy based formulas especially those that are rice-fortified where the highest As concentrations have been reported [12,13]. Rice and rice-based products are a reported source of i-As since they contain higher i-As concentrations compared to other foodstuffs, and are widely used during weaning, and to feed young children, due to its availability, bland taste, nutritional value and relatively low allergic potential [7,9,14–16]. Therefore, the EU has regulated the maximum levels of i-As in rice since January 2016 [17]. The most restricted level has been set at 0.1 mg/kg for rice-based foods destined for infants and young children and, in 2016, this threshold for rice destined for the production of food for infants and young children was proposed by the United States (US) [18]. In spite of these concerns regarding As in infants’ food, there is limited information available regarding infants’ dietary i-As exposure before weaning, and the impact of the EU i-As regulation in rice on infants’ i-As exposure once starting to consume solid foods, particularly for low i-As drinking water regions.

In this study urinary As speciation was carried out to assess the exposure levels in infants that were either breast or formula fed before weaning and after weaning. Moreover, As speciation was determined in rice-based products destined for infants and young children commonly used during weaning. The i-As levels of these foods were compared with the EU i-As standard, and discussed with the i-As concentrations previously reported in the same rice-based products analysed before the EU standard enforcement.

## Materials and methods

### Infant urine samples and sample preparation

The Office for Research Ethics Committees Northern Ireland (ORECNI; reference 14/NI/0047), and the Belfast Health and Social Care Trust approved the protocol of the study. Participants (mothers) provided informed written consent to participate. Mothers consented on behalf of the infants in a written consent. The protocol was designed to look at nutritional factors throughout pregnancy and into the neonatal period. Pre- and post-weaning urine samples were collected from infants from Belfast, Northern Ireland. Infants were enrolled at birth and a subset was followed up after weaning. Infant’s mothers met the inclusion criteria of ≥19 years old, non-smoker, white Caucasian, healthy nutritional status, and delivery scheduled at the reference hospital (Royal Jubilee Maternity Hospital, Belfast Health and Social Care Trust). About 70% of mothers were classified in the upper socioeconomic status, which included managers, senior technical staff, freelance professionals, intermediate occupations and managers in commerce. The infants included in the study were 41 girls and 38 boys, born in 2015, and classified according to the feeding mode before weaning as breastfed (n = 20), formula fed (n = 32), and partially breastfed (mixture of both methods) (n = 27). Information regarding pre-weaning infants’ rice-based formula consumption was not available. The pre-weaning urine samples were collected from infants with a median and range of 3.4 (1.3–6.7) months old. In a subset of 11 of these infants, born in September/October 2015, with a median and range of 2.1 (1.3–6.5) months old at pre-weaning sample collection, in addition to the pre-weaning samples post-weaning follow-up urine samples were collected once they reached a median and range of 7.7 (6.6–9.3) months old. At that time an interview with their mothers confirmed that 91% of the infants consumed rice or any rice-based product as part of their weaning diet.

Single spot urine samples, which are considered a good As exposure biomarker as opposed to 24-h or first morning void [19], were collected from the infant study population at home using cotton pads with As species content tested in blank samples, and clinically used for infants’ urine samples collection (Unisurge International Limited). The same type of cotton pad was used throughout the study, and they were provided along with detailed usage instruction before a scheduled visit at the infants’ reference pediatric clinic at Royal Jubilee Maternity Hospital, Belfast. If home collection was not possible, the infants’ urine sample was collected at the reference hospital during the scheduled visit. The urine was squeezed from the cotton pads, divided into aliquots, and frozen at or below -20ºC until analysis. Then, they were centrifuged with a Sorvall Legend RT at 4,500 rpm/2,200 g and 10 μl of analytical grade hydrogen peroxide (Normapur 30% H_2_O_2_) was added to 1 ml of sample to convert any arsenite to arsenate, to facilitate subsequent chromatographic detection as described in our previous publication [20]. The same procedure was applied for the blank cotton pad samples prepared by soaking them with ultrapure water or urine with known arsenic species content. Replicate samples of the certified reference material (CRM) urine ClinChek^®^—Control level I and blank samples were included in each analysis batch as quality control. The urine samples included in this study were not dilution adjusted. Solid food introduction into infant’s weaning diet may affect urine dilution, however, only minor changes were expected with a negligible effect on the results. Little benefit has been suggested from urine adjustment in previous studies, which have reported a strong correlation between adjusted and non adjusted urine samples with no significant differences in urinary As concentration, and disqualified the creatinine adjustment method due to association with the level of urinary As [21–23].

Tap water in Belfast is a uniform supply with reported low As concentration [24]. However, in order to provide a more descriptive As drinking-water level of exposure for the participants in this study, 3 tap water samples from Belfast, a city that has a single water supply source, were analysed for As speciation following the procedure described earlier for the urine samples.

### Rice-based infant products and sample preparation

Based on market availability a representative number of baby rice (n = 13), rice crackers/cakes (n = 29) and rice cereal (n = 31) samples belonging to 9 different most popular commercial brands or manufacturers were purchased from 17 food shops including online shopping in Belfast, Northern Ireland. Replicate samples of the sample products and brands were purchased from different stores or from the same store, for a few rice-cereal samples, but with different batch code (**S1 Table**). The rice-based product samples were collected in February 2016 and their use by dates were between June 2016 and November 2017, covering the whole weaning period for the children followed up in this study.

A Christ Alpha 1–4 LD Plus was used to freeze-dry all samples of rice-based products, and a Retch PM100 mill to powder them. Powdered sample, 0.1 g, was accurately weighed into 50 ml polypropylene centrifuge tubes and 10 ml of 1% concentrated nitric acid (Aristar 69% HNO_3_) was added and left overnight. A CEM MARS 6 was used to microwave digest the samples, and a Sorvall Legend RT to centrifuge them at 4,500 rpm/2,200 g. Then, a 1 ml aliquot was transferred to a 2 ml polypropylene vial and 10 μl of analytical grade hydrogen peroxide (Normapur 30% H_2_O_2_) was added to convert any arsenite to arsenate to facilitate subsequent chromatographic detection. Replicate samples of the CRM NIST 1568b rice flour and blank samples were included in each analysis batch as quality control. Further details regarding rice-based product samples preparation can be found in our previous study [7].

### Chemical analysis

Ion chromatography with IC-ICP-MS was used to analyse As speciation in urine, rice-based products, and water samples. It was a Thermo Scientific IC5000 ion chromatography (IC) system, with a Thermo AS7, 2x250 mm column and a Thermo AG7, 2x50 mm guard column interfaced with a Thermo ICAP Q ICP-MS in collision cell mode. A linear gradient mobile phase was carried out over 15 minutes starting at 100% mobile phase of 20 mM ammonium carbonate and finishing at 100% mobile phase of 200 mM ammonium carbonate. The resulting chromatogram was compared with that for authentic standards; DMA, i-As, MMA, tetratmethylarsonium (TETRA) and arsenobetaine (AsB). DMA concentration series were used to calibrate the As present under each chromatographic peak. Further details regarding chemical analysis can be found in our previous studies [7,25]

### Statistical analysis

The As species concentrations were natural log transformed before studying the effect of the feeding mode on As species concentrations in infants’ urine before weaning, and the effect of the weaning process on urinary As species concentrations by applying analysis of variance (ANOVA)—Tukey’s range test, and the paired t-test, respectively. All statistical analyses were conducted within the R statistical package, version 3.2.3 [26]. The limit of detection (LOD) was calculated as the mean of the blank concentrations plus three times the standard deviation of the blank concentrations multiplied by the dilution factor. The ½ LOD value was assigned for statistical analyses of the data when samples were below the LOD.

## Results

### CRM recoveries

The urine CRM ClinChek®—Control level I percentage recovery ± SE was 113 ± 2%, 95 ± 2%, 102 ± 2%, and 95 ± 2% for i-As, MMA, DMA, and AsB, respectively, based on N = 9. The rice CRM flour NIST-1568b percentage recovery ± SE was 99 ± 1%, 126 ± 2%, and 101 ± 3% for i-As, MMA, and DMA, respectively, based on N = 4 (**S2 Table**). The LOD for As speciation, calculated from DMA calibration, were 0.005 μg/l and 0.003 mg/kg for the urine and rice-based product samples, respectively. Consistent trace levels of As close or below the LOD were found in the blank samples squeezed from cotton pads soaked with ultrapure water, and no differences in the urinary arsenic species content were found in the urine blank samples squeezed from the cotton pads.

### Urinary As speciation

**Fig 1:**
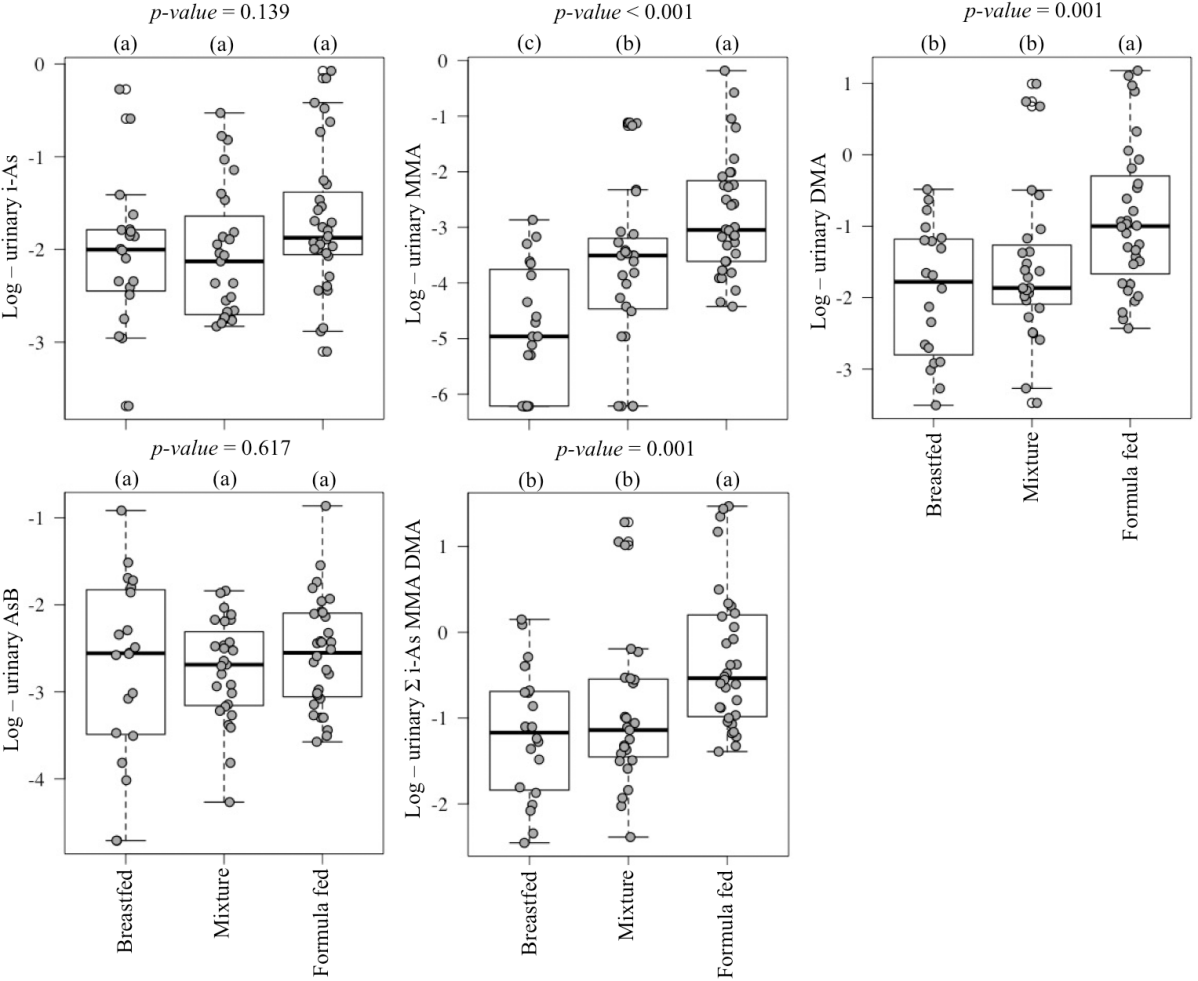
Urinary As species natural log concentrations according to the feeding mode before weaning (Values with the same letters were not significantly different at *p*-value <0.05 for the variable studied).

**Fig 2:**
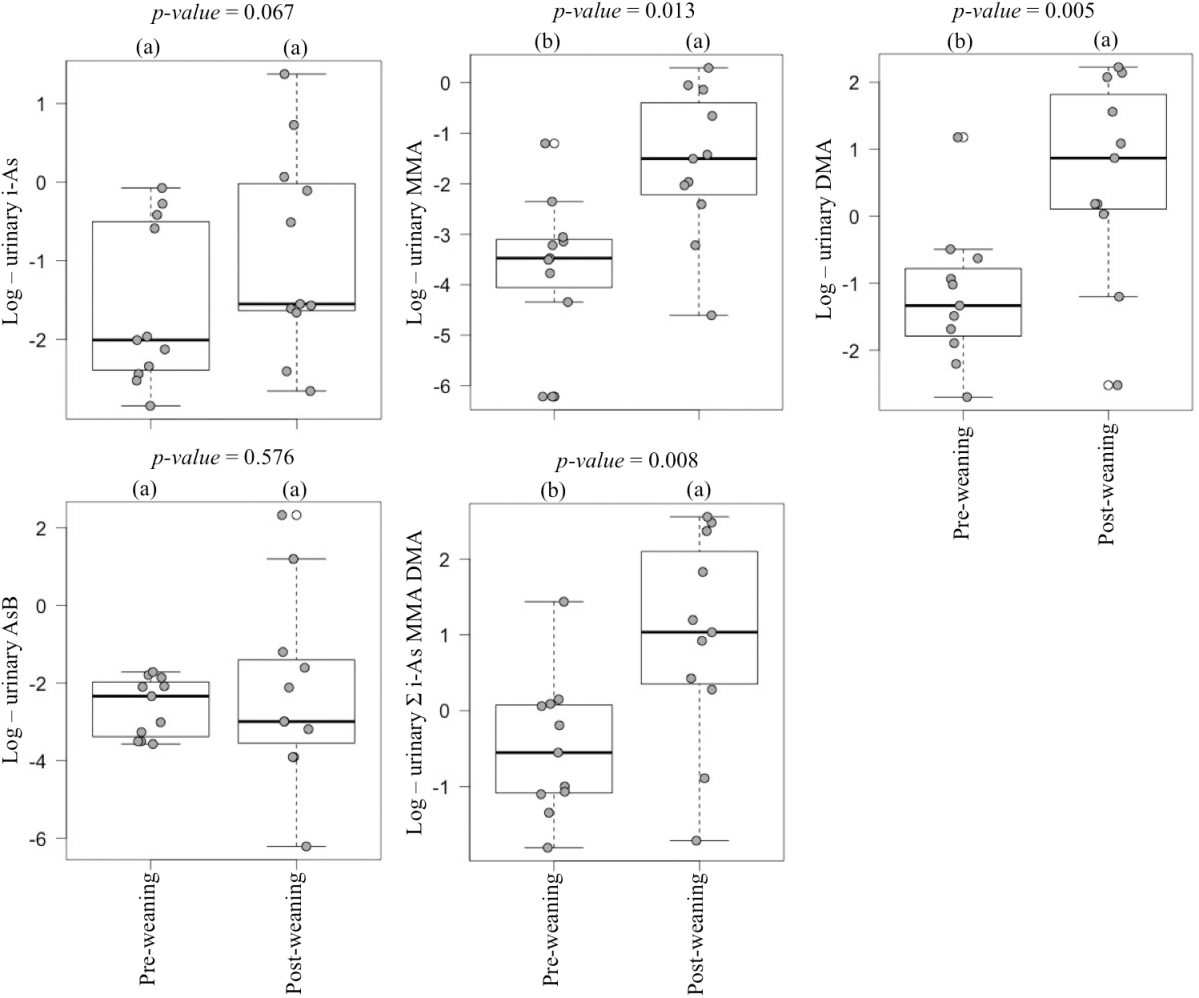
Urinary As species natural log concentrations in paired samples before and after weaning (Values with the same letters were not significantly different at *p*-value <0.05 for the variable studied).

All the urinary i-As, DMA, AsB, and 87% of MMA concentrations were higher than the LOD in the pre-weaning samples (n = 79). All the urinary i-As, MMA, DMA, and 91% of AsB concentrations were higher than the LOD in the post-weaning samples (n = 11). Total As (t-As) was calculated as the summation of i-As, MMA, and DMA, excluding AsB. Urine samples from pre-weaning infants, exclusively formula fed, had higher log MMA, log DMA and log t-As concentrations compared to those exclusively breastfed or partially breastfed (*p*<0.001, *p* = 0.001, *p* = 0.001, respectively) (**Fig 1**). The median urinary MMA, DMA, and t-As concentrations in pre-weaning urine from formula fed infants was 6.7-, 2.2-, and 1.9-fold higher compared to the levels found in samples from breastfed children, respectively (**Table 1**). Urine samples from post-weaning infants had higher log MMA, log DMA and log t-As concentrations compared to the paired pre-weaning samples (*p* = 0.013, *p* = 0.005 and *p* = 0.008, respectively) (**Fig 2**). The median urinary MMA, DMA, and t-As concentrations in post-weaning urine samples were 7.2-, 9.1-, and 4.8-fold higher compared to the level found in the paired pre-weaning samples (**Table 2**). The urine samples from post-weaning infants tended to have higher i-As concentration (*p* = 0.067), which was 1.6-fold higher than that for the pre-weaning urine paired samples (**Table 2** and **Fig 2**). No significant differences were found in urinary AsB concentration according to feeding mode before weaning or due to the weaning process (**Fig 1** and **Fig 2**).

**Table 1:** Urinary As speciation concentration (μg/l) and selected characteristics of the study population according to the feeding mode before weaning.

As species (μg/l)	Breastfed	Mixture	Formula
	n = 20	n = 27	n = 32
AsB	0.07 (0.01–0.40)	0.07 (0.01–0.16)	0.08 (0.03–0.42)
DMA	0.17 (0.03–0.61)	0.15 (0.03–2.70)	0.37 (0.09–3.25)
MMA	0.01 (0.00–0.06)	0.03 (0.00–0.32)	0.04 (0.01–0.83)
i-As	0.13 (0.02–0.76)	0.12 (0.06–0.59)	0.15 (0.04–0.92)
t-As^a^	0.31 (0.08–1.16)	0.32 (0.09–3.60)	0.58 (0.25–4.33)
Infant's age (months)	3.0 (1.9–6.0)	3.9 (1.3–6.7)	2.7 (1.4–6.1)
Sex (girls/boys)	7/13	15/12	19/13
Mother age (years)	33 (27–39)	31 (24–44)	32 (19–40)

Median (min–max).

^a^t-As = Σ(i-As + MMA + DMA).

**Table 2:** Urinary As speciation concentration (μg/l) and selected characteristics of the study population in paired urine samples pre- and post-weaning.

As species (μg/l)	Pre-weaning	Post-weaning
	n = 11	
AsB	0.09 (0.03–0.18)	0.05 (0.00–10.22)
DMA	0.26 (0.07–3.24)	2.38 (0.08–9.27)
MMA	0.03 (0.00–0.30)	0.22 (0.01–1.34)
i-As	0.13 (0.06–0.93)	0.21 (0.07–3.96)
t-As^a^	0.57 (0.16–4.21)	2.81 (0.18–12.89)
Infant's age (months)	2.1 (1.3–6.5)	7.7 (6.6–9.3)
Sex (girls/boys)	5/6	
Mother age (years)	29 (26–37)	

Median (min–max).

^a^t-As = Σ(i-As + MMA + DMA).

Tap water samples from the city of Belfast contained a median and range of i-As, DMA, and trimethylarsenic oxide (TMAO) concentrations of 0.042 (0.039–0.047) μg/l, 0.029 (0.027–0.031) μg/l, and 0.053 (0.050–0.057) μg/l, respectively.

### Rice-based products As speciation

All rice-based product samples had i-As, MMA and DMA concentrations higher than the LOD (n = 73). Up to 73% (n = 42) of baby rice and rice crackers specifically marketed for children exceeded the maximum i-As level of 0.1 mg/kg, ranging from 0.055 to 0.177 mg/kg with a median of 0.117 mg/kg. When including rice cereals in the calculations, packaging of which does not state that they are specifically for infants or young children, more than half of the rice-based products dataset analysed in this study (56%; n = 73) exceeded the maximum i-As standard with a median of 0.104 mg/kg. Almost 80% of the rice crackers, 61% of the baby rice, and 32% of the rice cereals exceeded the maximum i-As level established for rice destined for the production of food for infants and young children. Regarding baby rice samples, all Brand D and E samples exceeded the EU maximum i-As level with a median i-As of 0.147 mg/kg and 0.128 mg/kg, respectively. More than half of the baby rice Brand B samples analysed had i-As concentrations over 0.1 mg/kg, and only Brand A complied with the regulation with a median i-As of 0.056 mg/kg. All rice crackers belonging to Brand A and K, and almost all Brand E rice cracker samples, had higher i-As concentrations than 0.1 mg/kg with a median of 0.135 mg/kg, 0.153 mg/kg, and 0.117 mg/kg, respectively. Brand F and M rice cracker samples complied with the regulation with a median i-As of 0.086 mg/kg and 0.065 mg/kg, respectively. All Brand K, half of Brand F, and nearly one third of Brand J rice cereals i-As was over 0.1 mg/kg with median i-As concentration of 0.135 mg/kg, 0.090 mg/kg, and 0.081 mg/kg, respectively. Only Brand H rice cereal had lower i-As with a median of 0.078 mg/kg. The i-As concentration for the whole rice-based products dataset represented a median percentage of 74% of the summation of all As species, ranging from 46% to 86%. Thus, the i-As concentration was the predominant As specie in the rice-based products analysed followed by DMA, with a median and range concentration of 0.027 (0.013–0.109) mg/kg, and traces of MMA (**Table 3**).

**Table 3:** Concentration of As speciation (mg/kg) (median (min—max)) in rice-based products specifically marketed (baby rice and rice crackers) or consumed (rice cereals) by infants and young children ordered by type of product, commercial brand and year of collection.

Type of product	Commercial brand	Year	N	i-As (mg/kg)	DMA (mg/kg)	MMA (mg/kg)	% N > EU Regulation ^A^
Baby rice	All brands	2014	29	0.121 (0.056–0.268)	0.042 (0.023–0.123)	<LOD (<LOD—0.005)	58
		2016	13	0.103 (0.055–0.177)	0.080 (0.013–0.109)	0.006 (0.003–0.008)	61
	Brand A	2014	4	0.094 (0.088–0.097)	0.037 (0.024–0.041)	<LOD	0
		2016	2	0.056 (0.055–0.058)	0.013 (0.013–0.014)	0.008 (0.008–0.008)	0
	Brand B	2014	7	0.067 (0.056–0.092)	0.035 (0.030–0.061)	<LOD (<LOD—0.003)	0
		2016	7	0.101 (0.090–0.107)	0.097 (0.035–0.109)	0.005 (0.004–0.007)	57
	Brand C	2014	4	0.118 (0.062–0.129)	0.041 (0.023–0.046)	<LOD	75
	Brand D	2014	5	0.132 (0.122–0.142)	0.113 (0.107–0.120)	<LOD (<LOD—0.003)	100
		2016	3	0.147 (0.142–0.177)	0.067 (0.064–0.080)	0.004 (0.003–0.008)	100
	Brand E	2014	9	0.190 (0.117–0.268)	0.057 (0.038–0.123)	<LOD (<LOD—0.005)	100
		2016	1	0.128 (0.128–0.128)	0.063 (0.063–0.063)	0.007 (0.007–0.007)	100
							
Rice crackers	All brands	2014	36	0.127 (0.068–0.188)	0.026 (0.004–0.056)	<LOD	80
		2016	29	0.118 (0.063–0.165)	0.026 (0.014–0.063)	0.004 (0.003–0.005)	79
	Brand F	2014	2	0.132 (0.128–0.136)	0.021 (0.021–0.022)	<LOD	100
		2016	2	0.086 (0.074–0.098)	0.026 (0.026–0.027)	0.004 (0.004–0.004)	0
	Brand A	2014	6	0.127 (0.110–0.162)	0.024 (0.014–0.029)	<LOD	100
		2016	5	0.135 (0.117–0.162)	0.036 (0.028–0.060)	0.004 (0.003–0.004)	100
	Brand L	2014	3	0.096 (0.082–0.106)	0.036 (0.026–0.038)	<LOD	33
	Brand E	2014	25	0.132 (0.068–0.188)	0.026 (0.004–0.056)	<LOD	80
		2016	18	0.117 (0.090–0.165)	0.020 (0.014–0.033)	0.005 (0.004–0.005)	89
	Brand M	2016	2	0.065 (0.063–0.068)	0.062 (0.061–0.063)	0.005 (0.005–0.005)	0
	Brand K	2016	2	0.153 (0.152–0.154)	0.039 (0.039–0.040)	0.005 (0.005–0.005)	100
							
	All brands	2014	53	0.076 (0.008–0.323)	0.037 (0.005–0.082)	<LOD (<LOD—0.005)	18
		2016	31	0.081 (0.056–0.138)	0.025 (0.016–0.062)	0.004 (0.004–0.005)	32
Rice cereals	Brand F	2014	5	0.069 (0.066–0.123)	0.029 (0.024–0.037)	<LOD	40
		2016	2	0.090 (0.074–0.107)	0.030 (0.026–0.034)	0.004 (0.004–0.004)	50
	Brand G	2014	3	0.162 (0.104–0.167)	0.038 (0.016–0.052)	<LOD	100
	Brand H	2014	6	0.074 (0.044–0.097)	0.023 (0.005–0.035)	<LOD	0
		2016	2	0.078 (0.071–0.086)	0.024 (0.024–0.025)	0.004 (0.004–0.004)	0
	Brand I	2014	2	0.234 (0.146–0.323)	0.047 (0.026–0.069)	0.003 (<LOD—0.004)	100
	Brand J	2014	30	0.075 (0.008–0.188)	0.045 (0.013–0.082)	<LOD (<LOD—0.005)	10
		2016	25	0.081 (0.056–0.125)	0.025 (0.016–0.041)	0.004 (0.004–0.004)	28
	Brand K	2014	7	0.062 (0.033–0.096)	0.023 (0.010–0.036)	<LOD	0
		2016	2	0.135 (0.127–0.138)	0.057 (0.052–0.062)	0.004 (0.004–0.005)	100

^A^Percentage of samples exceeding the i-As EU maximum level for rice destined for the production of food for infants and young children. The 2014 data are from a previous study, and the 2014 rice crackers data only include those specifically marketed for infants and young children [7]

## Discussion

In this study infants who were fed exclusively with formula had higher exposure levels to As compared to those exclusively breastfed or partially breastfed as determined by a higher urinary concentration of DMA and MMA. Moreover, the weaning process increased the As exposure and infants post-weaning had higher urinary DMA and MMA concentrations compared to pre-weaning. Infants had access to very low As drinking-water (<0.1 μg/l, including i-As and DMA). Therefore, rice-based product consumption with high i-As concentration may play a predominant role in the infants’ dietary i-As exposure since more than 90% of them confirmed consuming these products during weaning.

Higher median t-As (0.41μg/l), excluding AsB, was found here for infants before weaning regardless of the feeding mode than that reported in breast and formula-fed infants at approximately 6 weeks of age from the New Hampshire Birth Cohort Study (NHBCS) in the US (0.17 μg/l), but lower compared to that reported in a highly As exposed population in Bangladesh (median of 1.2 μg/l, specific gravity adjusted, for breastfed and partially breastfed 3-months-old infants)[8,11].

Urinary As concentrations have been reported to be higher for formula fed infants compared to those exclusively breastfed (p < 0.0001) belonging to the NHBCS [8], and this study agrees with that. In addition, the study here goes further and provides information about urinary As metabolites, and reports that urinary DMA and MMA were affected by the feeding mode, concentrations of which were higher for formula fed infants compared to those breastfed. This increment of DMA and MMA in urine from infants formula fed may reflect i-As exposure since i-As is expected to be the main As specie in powdered formulas [6,27,28]. It also corroborates the early childhood ability to methylate i-As, which has been reported to be more efficient for children compared to adults [29]. Although further studies are needed to better evaluate infant’s ability to methylate i-As exposure it seems that formula fed infants had a stronger methylation ability, associated with a lower relative amount of urinary i-As, and higher relative amount of MMA and DMA, compared to those breastfed. DMA and MMA are the end metabolites of the i-As metabolism, however, it is important to bear in mind that reactive highly toxic intermediate metabolites such as DMA^III^ and MMA^III^ may be formed and pose health concerns [28].

Rice and rice-based products are common during the weaning process and when feeding young children, especially those suffering from celiac disease, however, they may contain high concentrations of i-As, and therefore maximum i-As levels have been established to protect the most vulnerable subpopulations such as infants and young children [7,17,30]. In this study, nearly ¾ of the baby rice and the rice crackers labelled specifically for infants and young children exceeded the EU maximum i-As limit (0.1 mg/kg). Rice-based product As content was dominated by i-As, however, a wide i-As concentration range was found (**Table 3**). Higher i-As content has been previously found in rice-based products containing brown rice compared to those manufactured with polished rice [7,31]. The rice growing origin has also been identified to affect rice i-As content in prior studies, and rice grown in the EU, characterised by a high relative amount of i-As, is expected to be predominant in the rice-based products included in this study [10,32]. Little changes were found in the i-As concentration in baby rice, rice crackers, and rice cereals compared to the same rice-based products previously tested in 2014 before the EU i-As standard in rice (**Table 3**) [7]. The Brand B baby rice suggested an increase in i-As concentration just above the maximum limit in comparison with the median level found in 2014. Also Brand E rice crackers, and Brand J and K rice cereal sample batches indicated an increment of the number of samples with higher i-As concentrations than the EU limit compared to the same sample batches analysed in 2014. No changes in the i-As concentrations were found in Brand D and E baby rice, and Brand A rice cracker samples before and after the regulation despite their high concentration over 0.1 mg/kg. Only Brand F rice crackers showed a reduction of i-As concentration below 0.1 mg/kg, complying with the maximum limit. The exceedance of the i-As standard emphasises the urgent need for low i-As rice used to manufacture infants’ and young childrens’ food, which could be obtained by applying novel ways of rice processing or by identifying low i-As rice growing regions [10,32,33]. Rice cereal packaging does not state that they are specifically for infants or young children and thus fall under higher concentration categories despite the fact that they are widely consumed by young children and may contain over 0.1 mg/kg of i-As as reported in the study here and others [7]. This also applies to other rice and rice-based products not specifically labelled for infants and young children, which contribute to the i-As exposure of this vulnerable subpopulation. Indeed, significantly higher urinary concentrations of DMA and MMA have been found here for children after weaning once 6–9 months old, which is the most common reported period to introduce rice-based products into young children’s diets [9], compared to those before starting consuming solid foods. This urinary increment of DMA and MMA may reflect i-As exposure and thus health risks as pointed out earlier. However, the methylated species may also be excreted in the urine unchanged lessening health risks, since noticeable levels of DMA and traces of MMA have been reported in food including rice and rice-based products in contrast with the very low As levels in the Northern Ireland drinking water found in this study [7]. Higher urinary DMA have been previously associated with rice consumption in children and adults [22,34]. It is worth mentioning the increment trend of urinary i-As found in children post-weaning despite the small sample size, which may be related to a highly toxic i-As fraction excreted in the urine unchanged [28]. Urinary As concentration has recently been associated with rice cereal and rice snack consumption in young children at 12 months belonging to the NHBCS in the US, where the 0.1 mg i-As/kg threshold for rice destined for the production of food for infants and young children is still under consideration, and i-As concentrations higher than that level have been reported in rice-based products [7,9]. They found that young children consuming rice-based products had an average of 2.5-fold higher urinary t-As compared to those that did not consume them. Urinary As speciation was also carried out in a subset of samples with rice consumption, concentration pattern of which was similar to that found here in post-weaning infants for i-As (0.24 μg/l) and DMA (3.00 μg/l), but lower MMA (0.92 μg/l) [9]. These high levels of urinary arsenic concentrations in infants post-weaning consuming rice-based products after the EU i-As regulation in rice suggests little impact of the regulation on infants’ i-As exposure, and indicates that efforts should be made to provide infants and young children with low i-As rice and rice-base products in order to protect this vulnerable subpopulation.

## Supporting information

S1 Dataset Urinary As speciation concentration (μg/l) according to the feeding mode used in Table 1 and Fig 1. (XLSX)

S2 Dataset Urinary As species concentrations (μg/l) in paired samples before and after weaning used in Table 2 and Fig 2. (XLSX)

S3 Dataset Concentration of As speciation (mg/kg) in rice-based products specially marketed or consumed by infants and young children bought in 2016 and used in Table 3. (XLSX)

S1 Table Rice-based infant products sampling details collected in 2016. (XLSX)

S2 Table Certified, found and recovery values for the certified references materials ClinChek®—Control level I, and Rice flour NIST-1568b. (XLSX)
